# Perioperative Low Dose Dexmedetomidine and Its Effect on the Visibility of the Surgical Field for Middle Ear Microsurgery: A Randomised Controlled Trial

**DOI:** 10.3389/fphar.2022.760916

**Published:** 2022-02-08

**Authors:** Jinhong Wu, Yuan Han, Yu Lu, Yan Zhuang, Wenxian Li, Ji’e Jia

**Affiliations:** Department of Anesthesiology, Eye & ENT Hospital, Fudan University, Shanghai, China

**Keywords:** dexmedetomidine, pharmacokinetics, pharmacodynamics, middle ear microsurgery, deliberate hypotension

## Abstract

**Background and Purpose:** There are many benefits of administering dexmedetomidine perioperatively. The pharmacokinetics (PK) and pharmacodynamics (PD) of intravenous, intranasal and oral dexmedetomidine that was administered before anesthesia were compared in this study, and the effects of dexmedetomidine on the surgical field visibility in tympanoplasty was evaluated.

**Methods:** A single-blind, randomized controlled trial was conducted in a university-affiliated hospital where 45 patients who underwent tympanoplasty under general anesthesia were randomly allocated into three groups. Dexmedetomidine was administered by intravenous infusion at 0.8 μg.kg^−1^ for 10 min, intranasal instillation at a drop rate of 1 μg.kg^−1^ and oral intake at 4 μg.kg^−1^ ten minutes before the induction of anesthesia. The PK and PD of dexmedetomidine after a single low dose administration and its effect on the surgical field in tympanoplasty were analysed.

**Results:** A plasma concentration of dexmedetomidine of 220 pg/ml was achieved immediately after intravenous infusion and at 13.2 and 70.3 min for intranasal and oral administration, respectively. Dexmedetomidine decreased the heart rate (HR) and mean arterial pressure (MAP) in all three groups, although these values remained higher in the oral dexmedetomidine group at all eight time points. Intravenous dexmedetomidine provided the best visualization of the surgical field for opening of the tympanic sinus, 30 min after the start of the infusion (*p* < 0.05). Intranasal dexmedetomidine provided a significantly better visual field than oral dexmedetomidine for the repair of a tympanic membrane perforation using the fascia temporal muscle (*p* < 0.05).

**Conclusion:** A single low dose of dexmedetomidine administered intravenously or intranasally could decrease HR and MAP, improve surgical field visibility and be appropriate for deliberate hypotension for surgical procedures of 1–2 h in length.

**Trial registration:** Clinicaltrials.gov identifier: NCT03800641.

## Introduction

Dexmedetomidine is an α2-adrenoceptor agonist with sedative, anxiolytic, analgesic and sympatholytic effects (Weerink et al., 2017; Pan et al., 2019), with its perioperative infusion improving the survival of patients undergoing cardiac surgery by five years (Peng et al., 2021). The effective range of plasma concentration of dexmedetomidine was reported to be 0.22–2.50 ng/ml for patients in the intensive care unit (Fujita et al., 2013). The pharmacokinetics (PK) of intranasal and intravenous dexmedetomidine have been described (Iirola et al., 2011; Li et al., 2018) but no PK data is available for oral dexmedetomidine. The maximal concentration (C_m_) of dexmedetomidine in the plasma is less than 0.1 ng/ml after 2 μg/kg was administered orally (Anttila et al., 2003). The delineation of the plasma concentration of dexmedetomidine is important, as the effects of the drug vary according to its plasma concentration. At high concentrations, dexmedetomidine increases the mean arterial pressure (MAP) and decreases the heart rate (HR). Meanwhile, at low plasma concentrations, dexmedetomidine may reduce MAP and HR by activating presynaptic α_2_-adrenoceptors in the central nervous system and vascular endothelial cells, causing vasodilation (Colin et al., 2017) which may have a potential role in deliberate hypotension.

Recently, the bioavailability of intranasal dexmedetomidine in adults administered using an atomizer at a drip rate of 60 drops/min was estimated to be 40.6%, with a mean onset of effect at 47.5 min after administration (Li et al., 2018). The absorption following the intranasal administration of drugs is rapid; for example, the time to C_m_ (T_m_) for intranasal midazolam is 10.9 (6.0–24.0) min (Schrier et al., 2017), while the T_m_ of oral midazolam is 42 ± 24 min (Reed et al., 2001). Similarly, in children, the plasma concentrations of dexmedetomidine reached 100 pg/ml within 20 min of atomized intranasal administration of 1 μg/kg dexmedetomidine (Miller et al., 2018). An earlier study reported a bioavailability of 65% for intranasal dexmedetomidine, with a T_m_ of 38 min (Iirola et al., 2011). Therefore, the PK of intranasal dexmedetomidine remains to be fully elucidated, with a higher C_m_ for intranasal administration of dexmedetomidine in the supine position. Moreover, an appropriate dose of oral dexmedetomidine has not been reported.

Controlled hypotensive anesthesia using dexmedetomidine has been reported to decrease intraoperative bleeding and enhance the quality of the surgical field (Snidvongs et al., 2015; Qiao et al., 2016). For successful microsurgery of the middle ear, a bloodless operative field is essential to improve surgical outcomes (Gupta et al., 2017). Good visibility of the surgical field is required at all time points of middle ear surgery, including skin incision, bone drilling, clearance of cholesteatoma and laying of the fascia (Yuan et al., 2019). However, the role of a single low dose of dexmedetomidine administered by a different route in achieving deliberate hypotension for middle ear microsurgery remains elusive. Four hours after the intramuscular administration of dexmedetomidine, the MAP and HR declined by 20 and 10%, respectively (Li et al., 2018), providing appropriate visibility during functional endoscopic sinus surgery.

This study aimed to investigate the PK and pharmacodynamics (PD) of intravenous, intranasal and oral dexmedetomidine and to explore the effects of dexmedetomidine on improving the visibility of the surgical field for middle ear microsurgery.

## Methods

### Ethics Approval

The SPIRIT statement (Chan et al., 2013) was adhered to in the study protocol which was approved by the Ethics Committee of the Eye & ENT Hospital of Fudan University. The trial was registered with ClinicalTrials.gov (NCT03800641) and was conducted in accordance with the International Conference on Harmonization Guidelines for Good Clinical Practice and the Declaration of Helsinki. The reporting of the study adhered to the CONSORT statement (Schulz et al., 2010).

### Study Design, Setting and Population

This single-blind, single-institution, randomized controlled trial in a university-affiliated hospital enrolled adult patients aged between 18 and 60 years old, who underwent tympanoplasty under general anesthesia between 11 January 2019 and 23 January 2020. Patients who had an American Society of Anesthesiologists (ASA) grade I physical status were eligible for the study. Patients with cardiorespiratory, hepatic or renal disease and those who had received dexmedetomidine within the previous week were excluded. Patients with acute upper respiratory tract infections or gastroesophageal reflux disease within one week of the study were also excluded. Prior to surgery, the patients fasted overnight according to the Buchinger guidelines with a daily caloric intake of 200–250 kcal, accompanied by a moderate-intensity lifestyle program.

## Study Procedures

### Consent and Randomization

The eligibility and interest in participating were assessed using a preoperative telephone call. Written informed consent was obtained from all participants. The patients were randomized and placed into one of three groups using computer-generated codes in a 1:1:1 ratio to receive intranasal (group I), intravenous (group II) or oral dexmedetomidine (group III) 10 min before the induction of anesthesia. In group I, dexmedetomidine (1 μg/kg, 10 μg/0.1 ml; Jiangsu Hengrui Medicine Co., Jiangsu, China) was introduced into the nasal cavity using a 1 ml syringe, with the patient seated on the operating table and the head tilted back. In group II, dexmedetomidine (0.8 μg/kg, 4 μg/ml) was infused *via* a peripheral intravenous cannula for 10 min. In group III, dexmedetomidine (4 μg/kg, 10 μg/0.1 ml) was administered orally in 5 ml of 5% glucose solution.

### General Anaesthesia

After dexmedetomidine administration as mentioned above, the patient was placed in the supine position with the head in the midline and general anesthesia was induced, by administering propofol (2–3 mg/kg), sufentanil (0.2 μg/kg) and cisatracurium (0.1 mg/kg). Anesthesia was maintained by sevoflurane at a minimum alveolar concentration of 1.3, and injection of sufentanil (0.1 μg/kg) and cisatracurium (0.05 mg/kg) every 45 min, and parecoxib sodium (1 mg/kg, maximum 80 mg) at the end of the operation for postoperative analgesia. Blood pressure was managed by an experienced anesthesiologist, with the target for controlled hypotension set for a 15% reduction *versus* baseline MAP. A flexible laryngeal mask airway (Teleflex, Morrisville, NC, United States) was inserted using the index finger technique by an anesthesiologist with at least 4 years of experience in endotracheal intubation. Mechanical ventilation was performed using a Primus anesthetic system (Dräger, Lübeck, Germany), with the following ventilation parameters: pressure-controlled ventilation mode, ventilation pressure 10–15 cm H_2_O, respiration rate 12/min, P_ET_CO_2_ (35–45 mmHg) and oxygen concentration 50%.

## Outcomes

### Patient Evaluation

The vital signs were recorded immediately before dexmedetomidine was administered, and were monitored intraoperatively for 2 h postoperatively by continuous electrocardiography and pulse oximetry. The HR and MAP were recorded immediately before and at 10, 20, 30, 45, 60, 90 and 120 min after dexmedetomidine administration. Bleeding within the intraoperative surgical field was evaluated using a scoring scale as described previously (Boezaart et al., 1995). The surgical field was evaluated 30 min after the start of surgery when the tympanic sinus was incised and 60 min after the start of surgery when the stapes-prosthesis was implanted. The perforation of the tympanic membrane was repaired using the fascia temporal muscle. The evaluators and surgeons were blinded to the patient’s group assignment.

### Pharmacokinetics Assays

The arterial blood samples were collected immediately before the induction of anesthesia, at 10, 20, 30, 45, 60, 90 and 120 min after dexmedetomidine administration. Plasma was separated by centrifugation and was stored at −20°C until batch analysis was performed. The plasma concentration of dexmedetomidine was determined by ultra-high performance liquid chromatography (UHPLC) using an Agilent 1290 Infinity™ II LC System (Agilent Technologies, Santa Clara, CA, United States) coupled with an Agilent 6470 Triple Quadrupole LC/MS (Agilent Technologies) at Zhipu Pharmaceutical Technology Co. (Shanghai, China) and analyzed using customized MATLAB software (Matlab, 2018a; MathWorks, United States). The technician was blinded to the patient data. Population PK modelling was performed as previously reported by Li et al. (2018) and Chamadia et al. (2020).

### Statistical Analysis

The sample size was calculated using PASS.12.0. According to previous studies, a difference of at least 1 in the surgical field evaluation score was considered clinically significant between groups. According to our preliminary study, the surgical field evaluation score had a standard deviation (SD) of 0.88. Assuming a power of 80% at a Bonferroni-adjusted alpha level of 0.025, the estimated sample size was 15 patients per group. The target population of 45 patients, with 15 patients in each group, was achieved.

Continuous data are expressed as mean ± standard deviation (SD)and compared using one-way analysis of variance (ANOVA). The evaluation of the surgical field was performed using the Kruskal-Wallis H test. All statistical analyses were performed using SPSS software (version 20.0; IBM, Armonk, NY, United States), with a *p*-value < 0.05 was considered statistically significant.

## Results

### Patient Demographic and Surgical Characteristics

**FIGURE 1:**
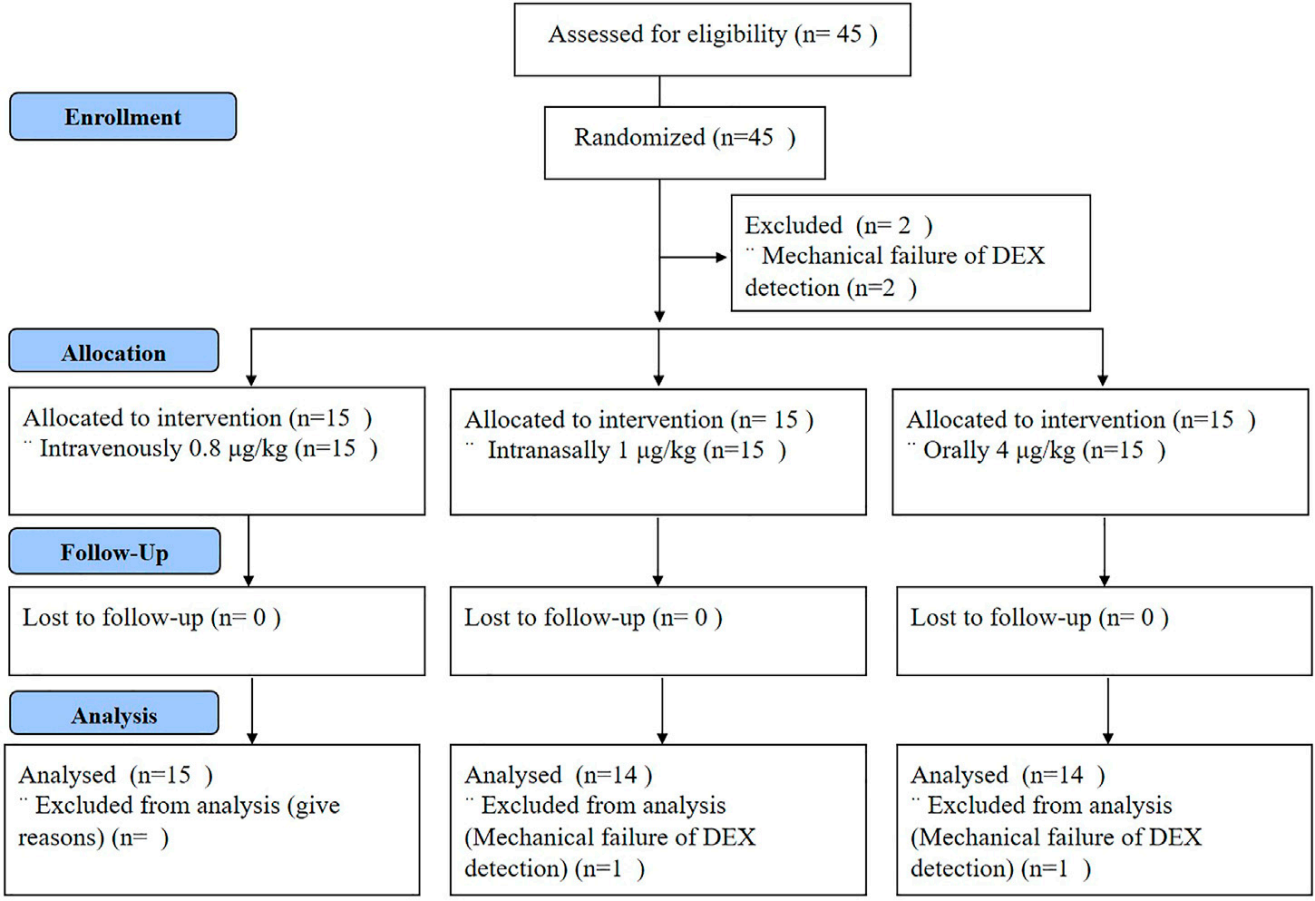
Study flowchart. A total of 45 patients were enrolled in this study and two patients were excluded. Plasma concentrations of DEX and clinical data were collected for 15 patients in the IV group, 14 in the IN group and 14 in the Oral group. DEX = dexmedetomidine; I.V. = intravenous; I.N. = intranasal.

A flowchart of the study is shown in Figure 1. Forty-five patients were included in this study having a median age of 40 years old (range 20, 59) and a median body mass index (BMI) of 22.01 kg/m^2^ (range 18.5, 24.9). Two patients without data on plasma dexmedetomidine concentrations were excluded from further analysis. The full analysis set included 43 patients, with 14, 15 and 14 patients in groups I, II and III, respectively. The demographic and surgical characteristics of the patients are shown in Table 1. It was reported that the participants in group II underwent a longer operative time but shorter recovery time than the other two groups (*p* > 0.05). In addition, the baseline HR was 73.53 ± 12.49, 75.57 ± 11.55, 76.36 ± 9.76 beats/min and MAP was 90.80 ± 12.51, 91.64 ± 8.85, 90.57 ± 10.69 mmHg in groups of I, II and III.

**TABLE 1:** Study subjects’ clinical characteristics.

	Group			Kruskal-Wallis test	ANOVA
	I.V.	I.N.	Oral		
Gender				*p* = 0.17	
Male	53.33%	35.71%	71.43%		
Female	46.67%	64.29%	28.57%		
Age	40.67 ± 11.54	42.57 ± 13.29	37.07 ± 11.76		*p* = 0.49
BMI	22.51 ± 2.21	21.59 ± 1.48	21.90 ± 2.32		*p* = 0.48
HR	73.53 ± 12.49	75.57 ± 11.55	76.36 ± 9.76		*p* = 0.79
MAP	90.80 ± 12.51	91.64 ± 8.85	90.57 ± 10.69		*p* = 0.96
Operation time (min)	103.67 ± 32.43	89.57 ± 17.79	96.71 ± 35.33		*p* = 0.45
Recovery time (min)	45.07 ± 14.20	48.29 ± 10.78	49.71 ± 11.97		*p* = 0.59
Propofol consumption (mg/kg)	2.23 ± 0.19	2.43 ± 0.25	2.36 ± 0.21		*p* = 0.07
Sufentanil consumption (μg/kg)	0.26 ± 0.04	0.25 ± 0.04	0.27 ± 0.06		*p* = 0.38
Sevoflurane consumption (ml/kg)	1.14 ± 0.52	0.84 ± 0.34	0.94 ± 0.36		*p* = 0.18

### Pharmacokinetics Characteristics of Dexmedetomidine

**FIGURE 2:**
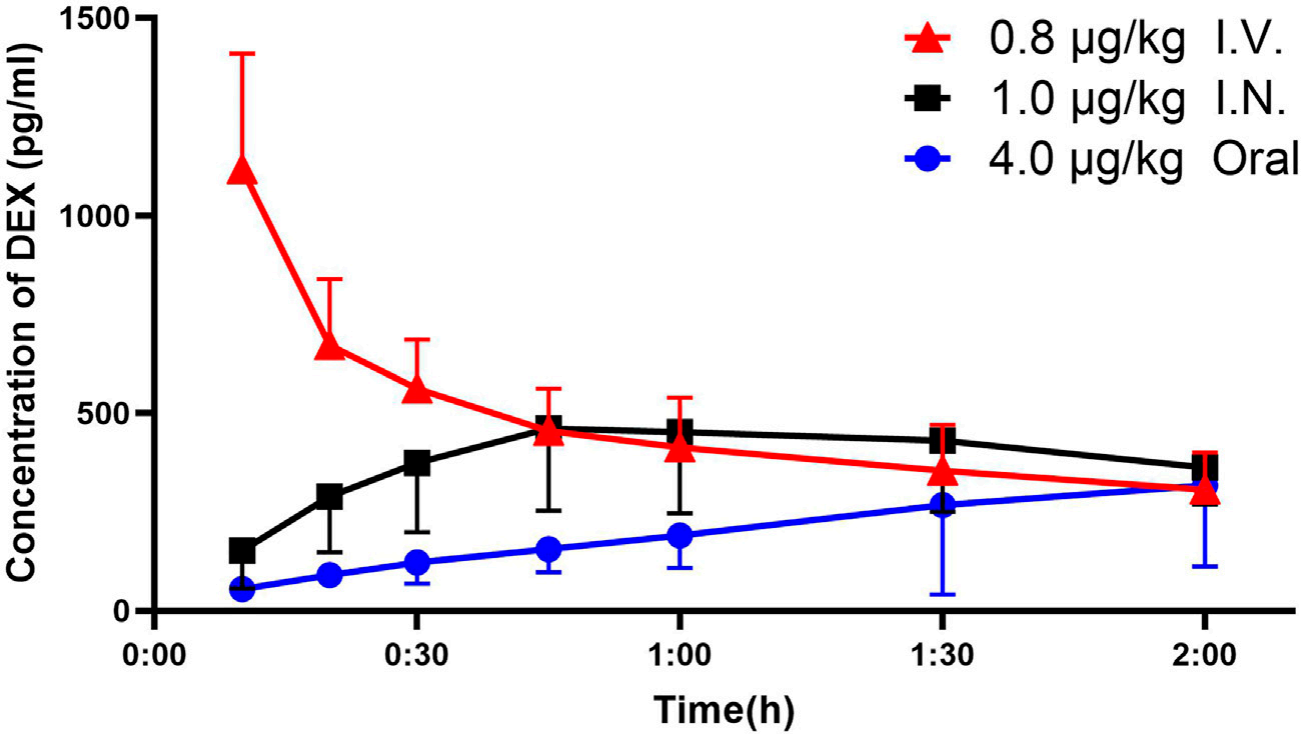
Plasma concentration–time curve and analysis of DEX. Plasma concentrations of DEX (mean ± standard deviation) were higher in the IV and IN groups at 10, 20 30, 45 and 60 min after administration compared to the oral group and higher in the IV groups at 10, 20 and 30 min compared with the IN group. DEX = dexmedetomidine; I.V. = intravenous; I.N. = intranasal.

**FIGURE 3:**
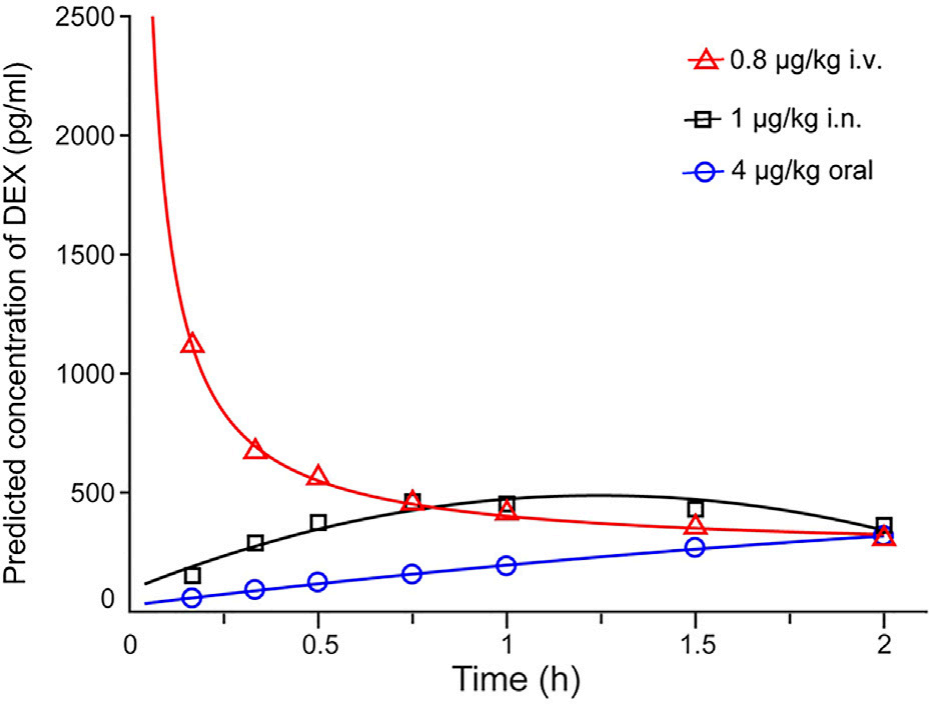
Predicted DEX plasma concentration–time curve. Plasma concentrations of DEX reached peak values after IV injection and decreased gradually, increased after IN infusion and slowly increased with oral administration. The time to achieve 220 pg/ml of DEX concentration was immediately after IV infusion, 13.2 min after IN and 70.3 min after oral administration. DEX = dexmedetomidine; I.V. = intravenous; I.N. = intranasal.

The actual and predicted time-concentration curves of dexmedetomidine after intravenous, intranasal and oral administration are shown in Figures 2, 3. Dexmedetomidine reached its C_m_ (220 pg/ml) immediately after intravenous infusion and rapidly declined, suggesting its rapid absorption and distribution. Dexmedetomidine gradually increased after intranasal instillation and the C_m_ was reached 13.2 min after administration. The orally-administered dexmedetomidine increased steadily and reached its C_m_ at 70.3 min post administration.

### Surgical Field Visibility

**FIGURE 4:**
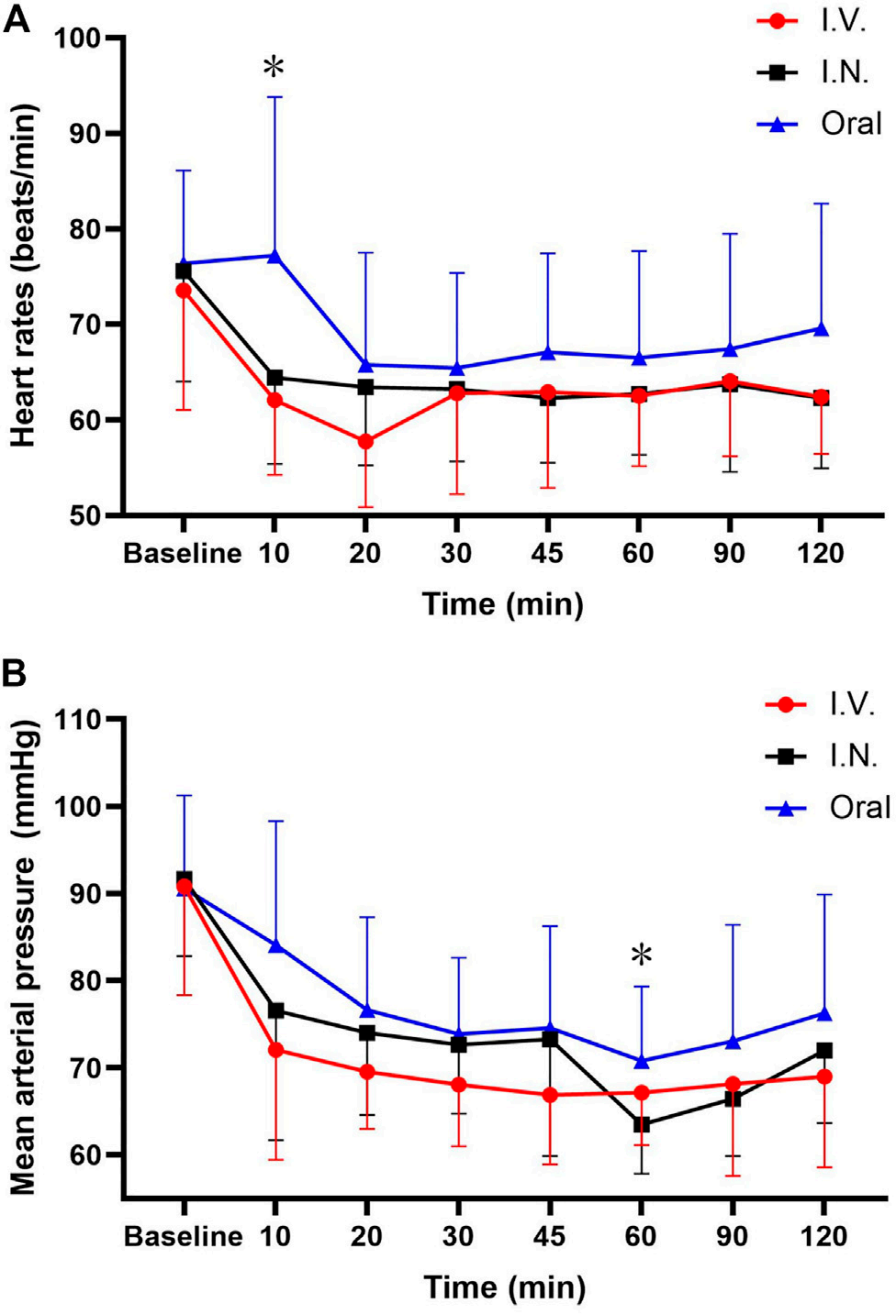
Analysis of HR and MAP after DEX administration. DEX administration decreased HR and MAP. The average HR and MAP were higher in group of oral administration at all eight time points. HR was significantly reduced after 10 min in the IV group compared with the IN and oral groups. MAP decreased remarkably after 60 min in the IN group compared to the IV and oral groups. *p < 0.05. DEX = dexmedetomidine; I.V. = intravenous; I.N. = intranasal.

Both HR and MAP declined after administration of dexmedetomidine and remained steady from 20 min after administration in all three groups (Figure 4). The mean HR and MAP were higher in group III than in groups I and II at all eight time points. HR was significantly reduced after 10 min in group II compared to groups I and III (*p* < 0.05). MAP was remarkably attenuated after 60 min in group I compared to groups II and III (*p* < 0.05).

The optimal surgical field at the time of incision of the tympanic sinus was obtained with intravenous dexmedetomidine compared to intranasal and oral dexmedetomidine (*p* < 0.05). At the time of repair of the tympanic membrane perforation using the fascia temporal muscle (60 min, *p* < 0.05), a better surgical field view was observed in the patients in group I than group III. The results of the surgical field visibility are presented in Table 2.

**TABLE 2:** Comparison of surgical field visibility.

Time (min)	Group	I.N.	Orally
30	I.V.	0.02*	0.01*
	I.N.	—	0.98
60	I.V.	0.41	0.23
	I.N.	—	0.04*

*p < 0.05.

## Discussion

Previous studies have reported the effects of dexmedetomidine on sedation, mild analgesia, inhibition of gland secretion and reduction of postoperative delirium (Subramaniam et al., 2019; Pereira et al., 2020; Qiu et al., 2020). In addition, dexmedetomidine is known to lower the dose of opioids and reduce the incidence of complications of general anesthesia, and recent research has revealed that sedation with dexmedetomidine might be reversed by d-amphetamine (Kato et al., 2021). Higher concentrations of dexmedetomidine are associated with greater sedation and analgesia, while decreasing HR and cardiac output (Ebert et al., 2000). Although dexmedetomidine is administered intravenously, intranasally, orally or subcutaneously in the clinical setting, the intranasal route is the most effective, well tolerated and convenient (Yuen et al., 2007). Comparative studies that focus on the PK and PD traits of dexmedetomidine administered *via* different routes are lacking.

The effects of intravenous, intranasal and oral administration of dexmedetomidine were compared by examining the concentrations of the different routes of administration of dexmedetomidine in this study. It was found that low dose dexmedetomidine improved the surgical field intravenously and intranasally, but not orally. The surgical field visibility improved markedly with the intravenous administration of dexmedetomidine compared to the intranasal or oral routes at the time of incision of the tympanic sinus (30 min after dexmedetomidine administration) and with intranasal dexmedetomidine than with oral dexmedetomidine at the time of repair of the tympanic membrane perforation with the fascia temporal muscle (60 min after dexmedetomidine administration). These findings are partly in line with a study which revealed that a single administration of 0.8 μg/kg dexmedetomidine improved surgical visibility and had a more favorable hemodynamic profile (Liu et al., 2016). The strength of this study is that clinically relevant plasma concentration levels were included.

When examining the correlation between plasma concentrations of dexmedetomidine and the surgical field score, it was considered that the surgical field score was not only related to the plasma concentration of dexmedetomidine at a certain time point, (such as the time when the tympanic sinus was incised) but to the continuous dexmedetomidine plasma concentration over time. Intravenous dexmedetomidine was associated with a plasma concentration that was continuously higher than 220 pg/ml 30 min before the tympanic sinus was incised and led to a clearer surgical field.

The reporting of plasma concentrations of dexmedetomidine in the clinical setting offers novel insights. Firstly, the onset time of the plasma concentration of dexmedetomidine after intranasal administration was approximately 15 min, which is significantly shorter than 47.5 min as previously reported (Li et al., 2018). This might be explained by the differences in patient positioning. A case report showed that the intranasal administration of dexamethasone drops in infants in the supine position could increase the risk of systemic absorption through the gastrointestinal tract, as this position would predispose to swallowing a significant portion of each drop administered (Orton and Censani, 2016). Secondly, there is evidence that oral dexmedetomidine had no effect on controlled hypotension due to the slow increase in plasma concentration, even less than 220 pg/ml by the end of the surgery.

The clinical data obtained in this study is in line with a recently published PK study where oral doses of dexmedetomidine between 300 and 700 mcg were associated with decreases in HR and MAP, while the T_m_ was approximately 120 min after oral intake (Chamadia et al., 2020). This indicates that dexmedetomidine *via* the oral route would have limited intraoperative clinical benefits for controlled hypotension. In addition, oral doses of dexmedetomidine may cause unexpected sedation or hemodynamic instability. The onset times of dexmedetomidine administered using by atomizers or drops differ significantly to other routes of administration (Li et al., 2016); the intravenous route has a significantly faster onset (15 min) than the intranasal route by atomizer (47.5 min) and drops (60 min) (Li et al., 2018). The present study clearly showed a T_m_ of approximately 45 min with nasal drops and a decline in MAP at 60 min.

There are some limitations to this study. Firstly, plasma concentration of dexmedetomidine to depict PK curves should have been obtained 8 h after administration. However, due to the limitation that all the participants were patients undergoing surgery, it is very difficult to obtain enough time points after surgery. Second, the sample size calculation did not increase the dropout rate. The secondary outcome is the difference of drug concentration between groups. While increasing the sample size needed to collect more patients’ blood samples. Therefore, for saving patient resources, we didn’t add the dropout rate. Third, we did not observe the sedation effect and complications of all groups, especially in the oral group, within the operating room and the post anesthesia care unit. Even though a low dose of dexmedetomidine was administered during this study, sedation was the most important effect. Therefore, future studies should focus on these aspects.

## Conclusion

Single low dose intravenous and intranasal dexmedetomidine can lower HR and MAP and improve visualization of the surgical field, which may be appropriate for deliberate hypotension for surgeries of 1–2 h in length. The visibility of the surgical field of the middle ear improved significantly with intravenous and intranasal administration of dexmedetomidine, compared to its oral administration.

## Data Availability

The original contributions presented in the study are included in the article/Supplementary Material, further inquiries can be directed to the corresponding authors.

## Abbreviations

PK, pharmacokinetics; Cm, maximal concentration; MAP, mean arterial pressure; HR, heart rate; Tm, the time to Cm; PD, pharmacodynamics; ASA, American Society of Anesthesiologists; SD, standard deviation; ANOVA, one-way analysis of variance.
